# Network Pharmacology Identifies Intersection Genes of Apigenin and Naringenin in Down Syndrome as Potential Therapeutic Targets

**DOI:** 10.3390/ph17081090

**Published:** 2024-08-20

**Authors:** Mohd Amir, Shabana Shafi, Shahida Parveen, Aijaz Ahmad Reshi, Ajaz Ahmad

**Affiliations:** 1 Department of Natural Products, College of Clinical Pharmacy, Imam Abdulrahman Bin Faisal University, Dammam 31441, Saudi Arabia; matahmad@iau.edu.sa; 2 Department of Computer Science, College of Computer Science and Engineering, Taibah University, Madinah 42353, Saudi Arabia; bhatshabu@gmail.com; 3 Department of Nursing, College of Pharmacy and Applied Medical Sciences (CPAMS), Dar Al Uloom University, Riyadh 13314, Saudi Arabia; sparveen@dau.edu.sa; 4 Department of Clinical Pharmacy, College of Pharmacy, King Saud University, Riyadh 11451, Saudi Arabia

**Keywords:** Down Syndrome, network pharmacology, apigenin, naringenin, molecular docking

## Abstract

Down Syndrome (DS), characterized by trisomy of chromosome 21, leads to the overexpression of several genes contributing to various pathologies, including cognitive deficits and early-onset Alzheimer’s disease. This study aimed to identify the intersection genes of two polyphenolic compounds, apigenin and naringenin, and their potential therapeutic targets in DS using network pharmacology. Key proteins implicated in DS, comprising DYRK1A, APP, CBS, and ETS2, were selected for molecular docking and dynamics simulations to assess the binding affinities and stability of the protein–ligand interactions. Molecular docking revealed that naringenin exhibited the highest binding affinity to DYRK1A with a score of −9.3 kcal/mol, followed by CBS, APP, and ETS2. Moreover, molecular docking studies included positive control drugs, such as lamellarin D, valiltramiprosate, benserazide, and TK216, which exhibited binding affinities ranging from −5.5 to −8.9 kcal/mol. Apigenin showed strong binding to APP with a score of −8.8 kcal/mol, suggesting its potential in modulating amyloid-beta levels. These interactions were further validated through molecular dynamics simulations, demonstrating stable binding throughout the 100 ns simulation period. Root mean square deviation (RMSD) and root mean square fluctuation (RMSF) analyses indicated minimal fluctuations, confirming the stability of the complexes. The findings suggest that apigenin and naringenin could serve as effective therapeutic agents for DS by targeting key proteins involved in its pathology. Future studies should focus on in vivo validation, clinical trials, and exploring combination therapies to fully harness the therapeutic potential of these compounds for managing DS. This study underscores the promising role of network pharmacology in identifying novel therapeutic targets and agents for complex disorders like DS.

## 1. Introduction

Down Syndrome (DS) is a genetic disorder caused by the presence of an extra copy of chromosome 21, known as trisomy 21 [1,2,3]. This chromosomal abnormality is most commonly detected in persons with intellectual disability, impacting approximately 1 in 700 live births [4]. DS is characterized by a range of physical and cognitive impairments, including developmental delays, intellectual disability, and distinctive facial features [2]. Individuals with DS also have a higher prevalence of congenital cardiac problems, respiratory and auditory impairments, Alzheimer’s disease (AD), and several autoimmune disorders [5,6]. Overexpression of genes located on chromosome 21 contributes to the intricate pathophysiology of DS [7,8]. The genes dual-specificity tyrosine-phosphorylation-regulated kinase 1A (DYRK1A), amyloid precursor protein (APP), cystathionine beta-synthase (CBS), and E26 transformation-specific transcription factor 2 (ETS2) play crucial roles in the cognitive and developmental impairments observed in individuals with DS [9,10,11]. DYRK1A is associated with deficits in neurodevelopment, while increased expression of APP results in an upregulation of amyloid-beta production, initiating the onset of early-onset AD [11,12]. CBS plays a crucial part in the metabolism of homocysteine, and any dysfunction in this process can lead to cardiovascular diseases and cognitive impairments [13]. ETS2 contributes to abnormal cell proliferation and apoptosis, hence increasing the complexity of DS pathogenesis [14]. Network pharmacology is an emerging discipline that integrates systems biology and pharmacology to offer a holistic understanding of drug mechanisms and interactions within biological networks [15,16]. Network pharmacology distinguishes itself from traditional pharmacology by considering the complex interplay of multiple targets and pathways, instead of merely focusing on individual target interactions [15]. This approach is particularly suitable for complex disorders like DS, where the advancement of the disease involves multiple genes and proteins [17]. Network pharmacology combines computational methods, bioinformatics, and systems biology to identify new treatment targets and understand the mechanisms by which bioactive molecules function. Researchers can utilize it to construct and analyze interaction networks, discern crucial nodes, and predict the ramifications of modifying these nodes [18]. This comprehensive method provides a deep understanding of the therapeutic potential of chemicals and their impacts on disease networks.

**Figure 1:**
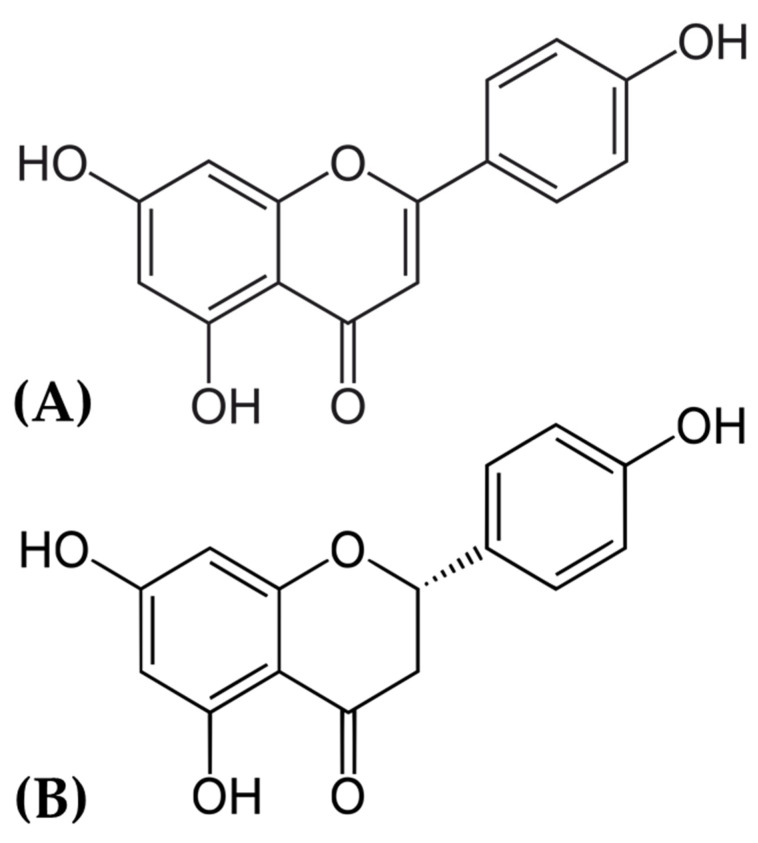
Chemical structure of (**A**) apigenin and (**B**) naringenin.

Previous studies have examined the therapeutic potential of several chemicals in DS using both laboratory-based and live organism models [2,19,20]. Various studies have demonstrated that polyphenolic compounds have the capacity to control significant pathways involved in DS, owing to their anti-inflammatory, antioxidant, and neuroprotective properties [21,22]. Studies have shown that polyphenols like resveratrol and curcumin can reduce oxidative stress and improve cognitive function in Down Syndrome (DS) mice [20,23,24]. Blocking the enzyme DYRK1A has been identified as a potential therapy strategy, as studies have shown that DYRK1A inhibitors can improve cognitive impairments in DS animals [25]. Apigenin and naringenin (Figure 1), two naturally occurring polyphenolic compounds, have garnered attention for their potential neuroprotective qualities [26,27,28,29]. Apigenin, found in various fruits and vegetables, has demonstrated anti-inflammatory, antioxidant, and potentially anticancer properties [29]. Naringenin, mostly found in citrus fruits, is well known for its anti-inflammatory and antioxidant characteristics and has shown potential in controlling metabolic disorders [30,31]. The aim of this study was to investigate the therapeutic potential of apigenin and naringenin in DS by identifying the shared genes and their potential targets using network pharmacology. The reason for selecting these polyphenols is based on their previously shown ability to preserve the brain and their power to control important pathways involved in neurodegenerative diseases [27,32]. The primary objective of this study was to ascertain the binding affinities and interactions between apigenin and naringenin and four crucial proteins linked to DS: DYRK1A, APP, CBS, and ETS2. The proteins were chosen based on their significant role in the pathophysiology of DS. The aim of our study was to employ molecular docking and molecular dynamics simulations to elucidate the stability and specificity of the interactions between these polyphenols and the target proteins. Molecular docking is a computer technique employed to determine the most favorable interactions between small compounds and their target proteins [33]. This technique provides useful insights about the possible efficacy of the drugs [33,34]. Molecular dynamics simulations further validate the enduring stability of these connections, improving our understanding of the dynamic behavior of the protein–ligand complexes [35,36]. This study employs network pharmacology to examine the treatment efficacy of apigenin and naringenin in DS. We aim to employ computational approaches and bioinformatics, in conjunction with experimental validation, to identify novel therapeutic targets and elucidate the mechanisms of action of these polyphenols. The findings of this study have the capacity to facilitate the development of effective treatment strategies for managing DS, showcasing the promise of network pharmacology in addressing complex hereditary disorders.

## 2. Results

### 2.1. Protein–Protein Interactions (PPI) Analysis

**Figure 2:**
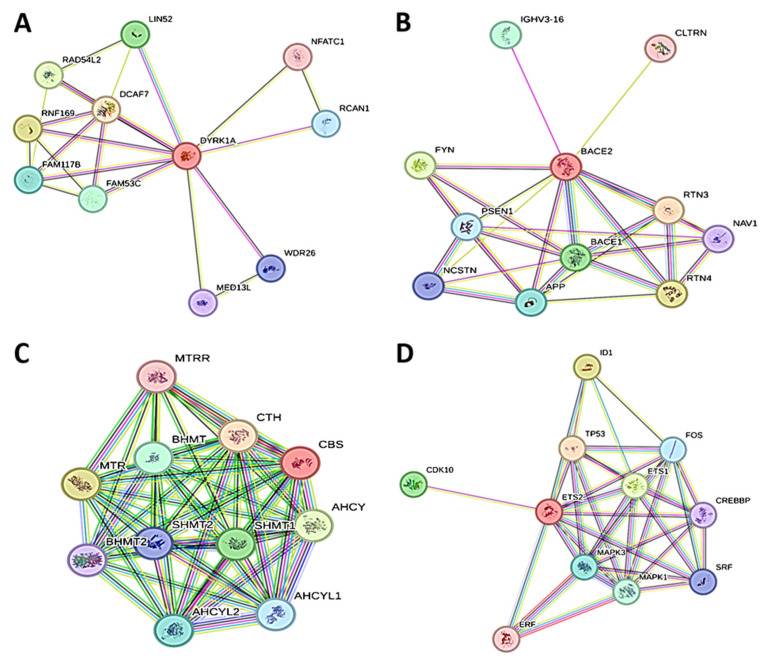
Protein–protein interactions of (**A**) DYRK1A, (**B**) APP, (**C**) CBC, and (**D**) ETS2.

The STRING database is a computational aspect that contains both anticipated and observed protein–protein interactions between possible therapeutic targets and other human proteins. The interactions are derived from a computer program estimation, knowledge transfer between organisms, and interactions aggregated from primary databases. They encompass physical and functional associations. The significance of protein–protein interactions (PPI) stems from its variety, adaptability, and precision. Studies on interactions between proteins have demonstrated an association between the targets (Figure 2). The DYRK1A, APP, CBC, and ETS 2 networks have 11, 11, 11, and 11 nodes and 22, 28, 53, and 35 edges, respectively. The higher nodes’ appearance indicates an increased level of connectedness. The circle-shaped structure denotes the network nodes that represent different proteins. Additionally, the red color demonstrates the query protein, and the stacked line shows the edges that represent the protein–protein association.

### 2.2. Molecular Docking Analysis

The primary goal of the molecular docking investigation was to identify the optimal polyphenol–protein interaction. To conduct molecular docking between the two polyphenols and their respective four proteins, PyRx tools AutoDock Vina wizard (V 1.2.5) was used. After molecular docking, the lead compounds’ binding affinities ranged from −7.2 to −9.3 kcal/mol, whereas the positive control drug’s binding affinities ranged from −5.5 to −8.9 kcal/mol (Table 1). Naringenin calculated the highest binding affinities of −9.3 kcal/mol and −8.0 kcal/mol with DYRK1A and CBS, respectively, whereas the apigenin showed a negative higher binding score of −8.8 kcal/mol and −7.3 kcal/mol with APP and ETS2, respectively. The positive control drug lamellarin D showed a binding score of −8.9 kcal/mol for DYRK1A, whereas valiltramiprosate, benserazide, and TK216 exhibited docking scores of −5.5, −6.4, and −7.1 kcal/mol for APP, CBS, and ETS2 proteins, respectively. The lead compounds apigenin and naringenin were calculated to have better native binding scores than the control drugs for every protein target.

### 2.3. Protein–Ligand Interaction Analysis from Molecular Docking

**Figure 3:**
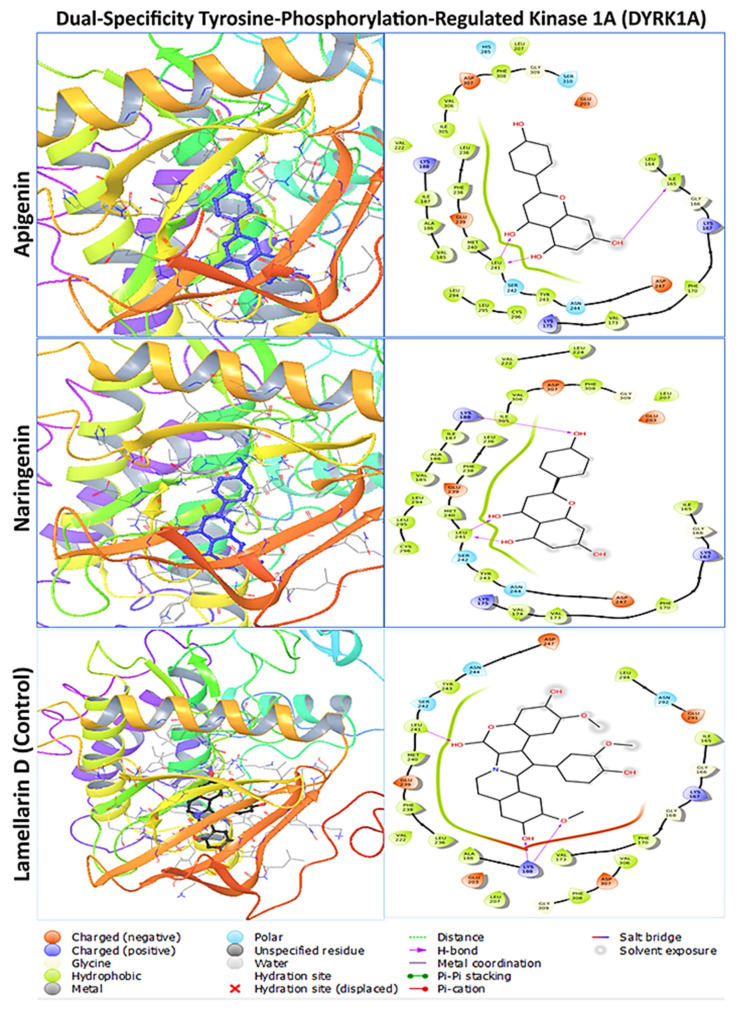
The molecular docking interaction of dual-specificity tyrosine-phosphorylation-regulated kinase 1A with two lead compounds of apigenin and naringenin and the positive control drug lamellarin D. The left side denotes the 3D protein–ligand structure and the right side denotes the formation of several bonds by the ligand complexed with protein.

**Figure 4:**
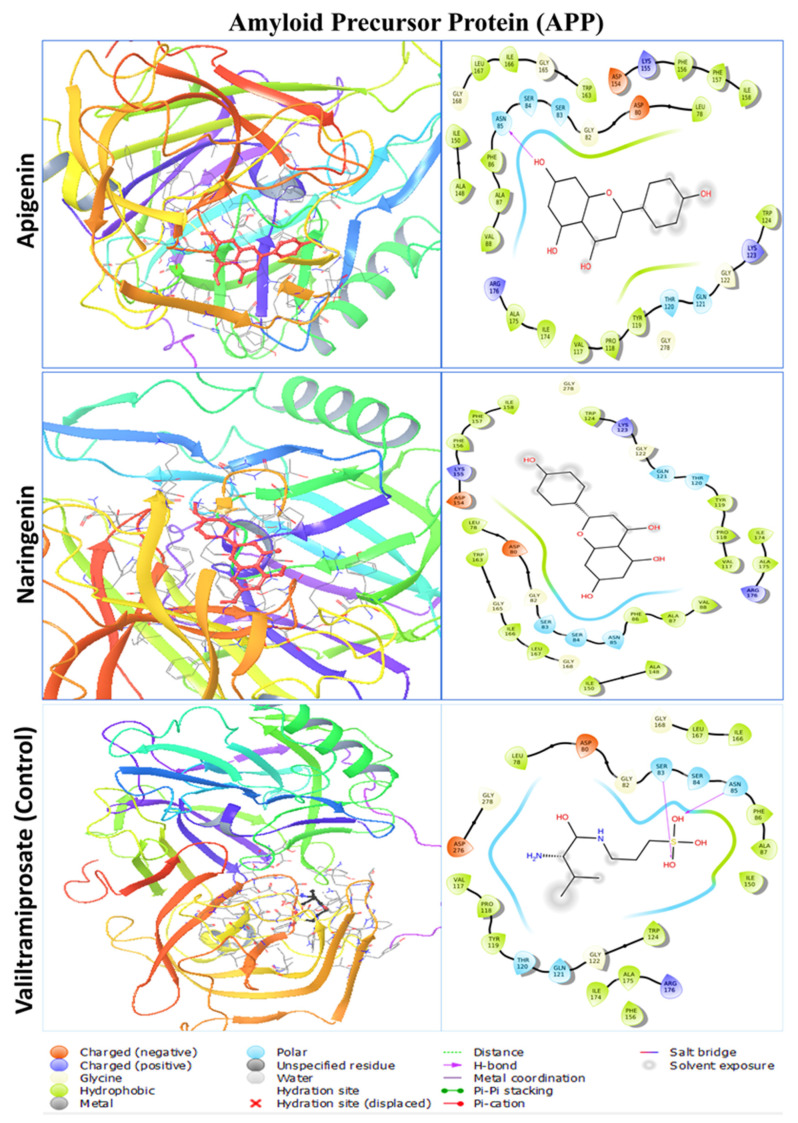
The molecular docking interaction of amyloid precursor protein with two lead compounds of apigenin and naringenin and the positive control drug valitramiprosate. The left side denotes the 3D protein–ligand structure and the right side denotes the formation of several bonds by the ligand complexed with protein.

**Figure 5:**
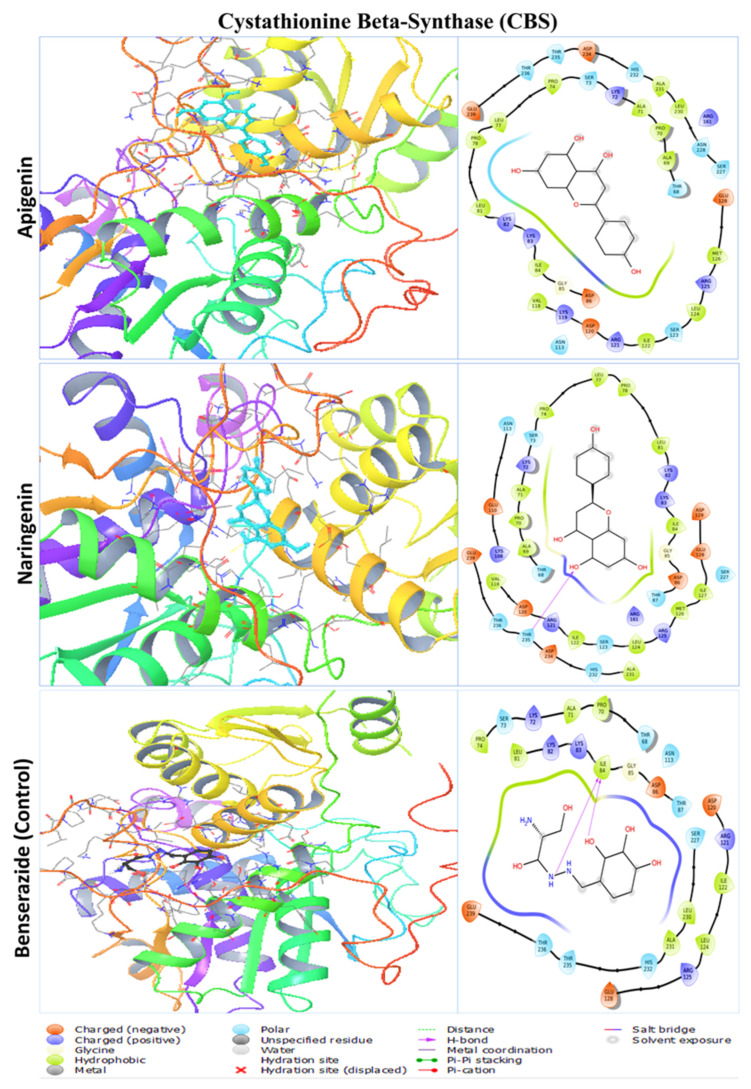
The molecular docking interaction of cystathionine beta-synthase with two lead compounds of apigenin and naringenin and the positive control drug benserazide. The left side denotes the 3D protein–ligand structure and the right side denotes the formation of several bonds by the ligand complexed with protein.

**Figure 6:**
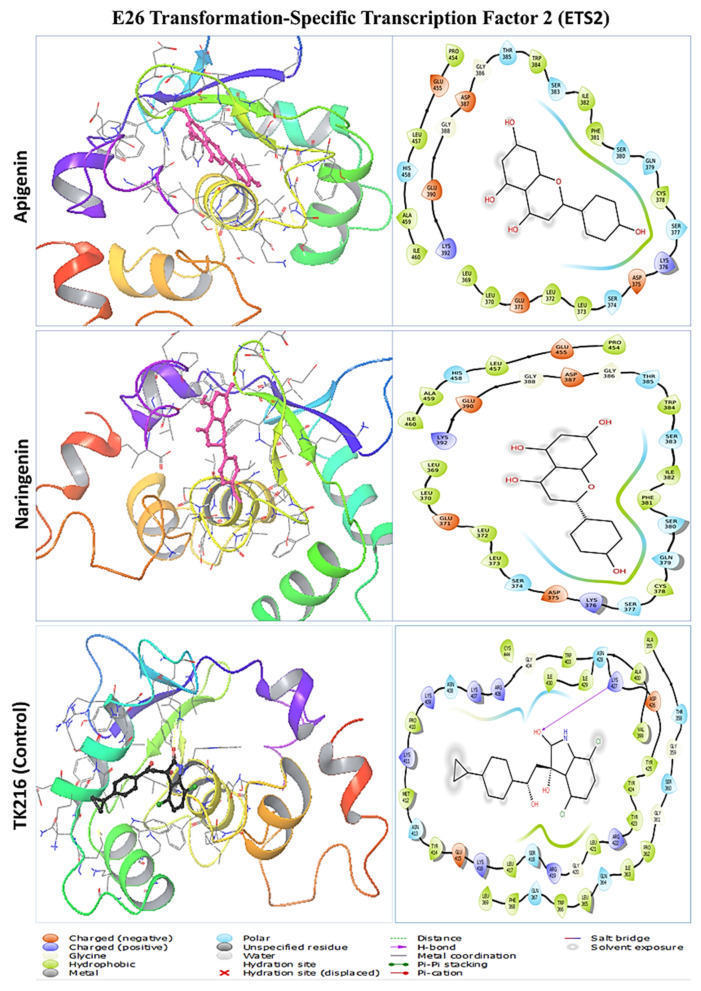
The molecular docking interaction of E26 transformation-specific transcription factor 2 with two lead compounds of apigenin and naringenin and the positive control drug TK216. The left side denotes the 3D protein–ligand structure and the right side denotes the formation of several bonds by the ligand complexed with protein.

The two polyphenols were retrieved for further analysis and molecular interactions were visualized through the Maestro module of the Schrodinger suite, as shown in Figure 3, Figure 4, Figure 5 and Figure 6 for the four protein–ligand complexes. Different types of non-bonded interactions between receptors and ligands, like hydrogen bonds, glycine bonds, metal bonds, electrostatic bonds, salt bridges, and hydrophobic bonds were generated and are depicted in Table 2. DYRK1A, ILE165, GLY166, LYS167, PHE170, VAL173, LYS175, VAL185, ALA186, ILE187, LYS188, GLU203, LEU207, VAL222, LEU236, PHE238, GLU239, MET240, LEU241, SER242, TYR243, ASN244, ASP247, HIS285, LEU294, LEU295, CYS296, ILE305, VAL306, ASP307, PHE308, and GLY309 are common interacting residues for both apigenin and naringenin. On the other hand, APP, LEU78, ASP80, GLY82, SER83, SER84, ASN85, PHE86, ALA87, VAL88, VAL117, PRO118, TYR119, THR120, GLN121, GLY122, LYS123, TRP124, ALA148, ILE150, ASP154, LYS155, PHE156, PHE157, ILE158, TRP163, GLY165, ILE166, LEU167, GLY168, ILE174, ALA175, ARG176, and GLY278 are common, for CBS, THR68, ALA69, PRO70, ALA71, LYS72, SER73, PRO74, LEU77, PRO78, LEU81, LYS82, LYS83, ILE84, GLY85, ASP86, ASN113, VAL118, ASP120, ARG121, ILE122, SER123, LEU124, ARG125, MET126, GLU128, ARG161, SER227, ALA231, HIS232, ASP234, THR235, THR236, and GLY239. ETS2, LEU369, LEU370, GLU371, LEU372, LEU373, HER374, ASP375, LYS376, SER377, CYS378, GLN379, SER380, PHE381, ILE382, SER383, TRP384, THR385, GLY386, ASP387, GLY388, GLU390, LYS392, PRO454, GLU455, LEU457, HIS458, ALA459, ILE460 are the common interacting amino acids that serves as active site for apigenin and naringenin while binding with specific target protein. The positive control drugs for each receptor also bound with multiple common interacting residues (Table 1) that suggested that the selected two ligands apigenin and naringenin are bound at the same active site of each receptor (Supplementary Figure S1). The DYRK1A protein control drug lamellarin D has similar binding amino acids positions at ILE165, GLY166, LYS167, PHE170, VAL173, ALA186, LYS188, GLU203, LEU207, VAL222, LEU236, PHE238, GLU239, MET240, LEU241, SER242, TYR243, ASN244, ASP247, LEU294, VAL306, ASP307, PHE308, and GLY 309, respectively, with the apigenin and naringenin binding pose. The APP protein control drug valiltramiprosate mutual binding site residues were LEU78, ASP80, GLY82, SER83, SER84, ASN85, PHE86, ALA87, VAL117, PRO118, TYR119, THR120, GLN121, GLY122, TRP124, ILE150, PHE156, ILE166, LEU167, GLY168, ILE174, ALA175, ARG176, ASP276, and GLY278, respectively. For the CBS protein control drugs benserazide, communal amino acids were THR68, PRO70, ALA71, LYS72, SER73, PRO74, LEU81, LYS82, LYS83, ILE84, GLY85, ASP86, THR87, ASN113, ASP120, ARG121, ILE122, LEU124, ARG125, GLU128, SER227, ALA231, HIS232, THR235, THR236, and GLU239, respectively, and the ETS2 protein control drug TK216 conjoint binding residues was LEU369 with two lead compounds.

### 2.4. Molecular Dynamic Simulation

The stability of protein–ligand complexes was confirmed in an artificial environment through MD simulation. To determine the stability of protein–ligand complexes, a 100 ns MD simulation was carried out. Results of MD simulations were described by using RMSD, RMSF, radius of gyration (Rg), and solvent-accessible surface area (SASA).

### 2.5. RMSD Analysis

**Figure 7:**
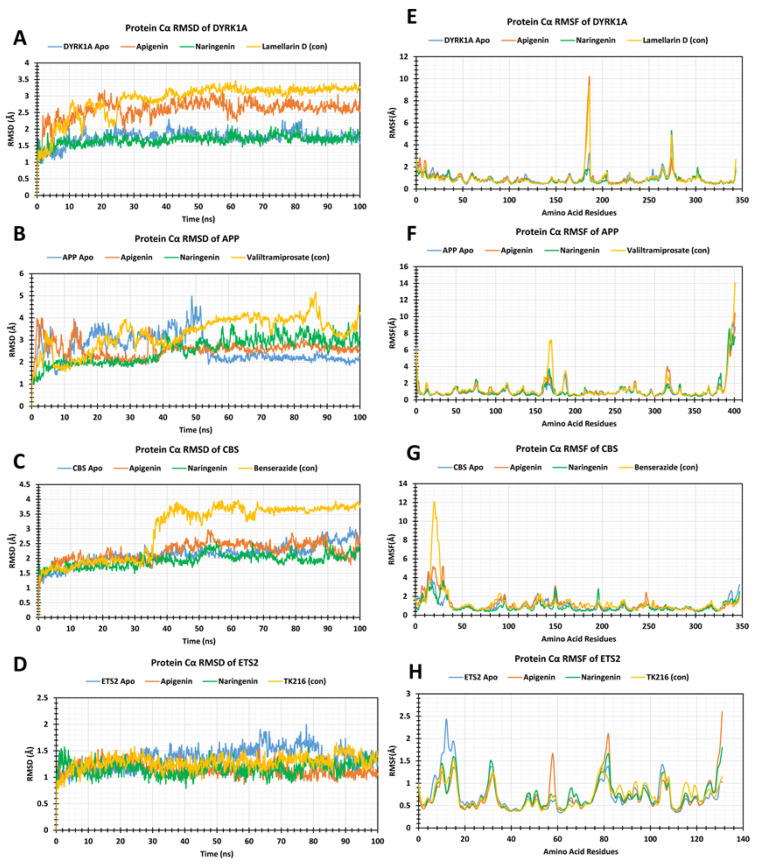
Graphs showing the protein carbon alpha of RMSD values (Left), and RMSF values (Right). The apo protein is depicted in blue, apigenin is marked orange, naringenin is marked green and the control drug for each receptor is shown in yellow. Apigenin and naringenin RMSD values when complexed with (**A**) DYRK1A, (**B**) APP, (**C**) CBC, and (**D**) ETS2, and apigenin and naringenin RMSF values when complexed with (**E**) DYRK1A, (**F**) APP, (**G**) CBC, and (**H**) ETS2.

The stability of the respective apo protein (negative control) of DYRK1A, APP, CBC, and ETS2, of each protein with the lead ligands (apigenin and naringenin), and respective protein drugs’ (positive control) complex structure was assessed by measuring the RMSD of the Cα atoms during the 100 ns simulation. For the DYRK1A receptor, DYRK1A Apo, the lead apigenin and naringenin in complex with DYRK1A, and the control drug lamellarin D showed average RMSD values of 1.73 Å, 2.58 Å, 1.67 Å, and 2.88 Å, whereas the lowest to highest RMSD values were (0.935–2.299) Å, (0.962–3.21) Å, (0.968–2.189) Å, and (0.912–3.466) Å, respectively, as demonstrated in Figure 7A. For APP, the average Cα-RMSD, and lowest-highest RMSD values of the APP apo, apigenin–APP, and naringenin–App, and control drugs APP–valiltramiprosate were 2.60 Å (0.936–4.979) Å, 2.57 Å (1.155–4.009) Å, 2.54 Å (1.043–3.775) Å, and 3.28 Å (0.934–5.174) Å, respectively. Notably, none of these compounds showed significant variations when compared with apo and control drug complexes, remaining in an equilibrium state throughout the simulation time frame (Figure 7B). In the case of CBS, the average and lower-higher deviations of CBS apo was 2.13 Å (1.06–3.06) Å, apigenin–CBS complex 2.21 Å (1.041–2.97) Å, naringenin–CBS was 1.92 Å (1.236–2.567) Å, and the control drug CBS–benserazide was 2.97 Å (1.083–3.97) Å, which demonstrates structural stability during the 100 ns simulation, in contrast to the instability (Figure 7C).

Similarly, for ETS2 apo, apigenin–ETS2, naringenin–ETS2, and ETS2–TK216 (control drug) complexes, as depicted in Figure 7D, the range was (0.83 to 1.992) Å, (0.868 to 1.545) Å, (0.79 to 1.74) Å, and (0.758 to 1.656) Å, respectively, indicating good stability, resembling the selected compound as promising lead agents when comparing with the positive control drug ETS2–TK216 drug complex. In all the four receptors of DYRK1A, APP, CBS, and ETS2, the apigenin and naringenin complexes showed better stability than all the comparable positive drug complexes. In DYRK1A, from the 0 ns to 100 ns simulation time frame, the apo protein and control drugs exhibited greater deviation. When the apigenin and naringenin were complexed with the apo protein, they showed less deviation that shown when the two lead compounds stabilized the protein structure. In case of APP, from 0 ns to 50 ns, all the structures depicted some random deviation; after that, the apigenin and naringenin provided the less RMSD values but the control drug showed a larger peak than the two lead compounds. The apigenin and naringenin complexed with the APP proteins demonstrated good stability. For CBS, the apigenin and naringenin complexes provided overlapping RMSD values with the negative control CBS–apo protein, whereas the positive control complex showed much higher RMSD values from the 35 ns simulation time to the 100 ns simulation time frame. Finally, for the ETS2 receptors, the two lead compounds’ complexes and control drug complex showed almost similar ranges of RMSD values from the start to almost the end of the 100 ns simulation time, but after the 85 ns simulation time, TK216 control drugs demonstrated a much higher peak than the lead compound complexes.

### 2.6. RMSF Analysis

The protein complexes with the selected polyphenols’ RMSF values are illustrated in Figure 7E–H. The apo protein, two lead ligands apigenin and naringenin, and the control drug lamellarin D in complex with DYRK1A exhibited average fluctuations of 0.95 Å, 1.00 Å, 0.846 Å, and 0.93 Å, respectively (Figure 7E). In the DYRK1A receptor, the apigenin exhibited GLN 320 (10.085 Å), the LYS 412 (5.279 Å), and the lamellarin D exhibited GLN 320 (9.198 Å), LYS 412 (4.923) Å residual positions have large peaks, whereas the naringenin compound did not show any major fluctuations in 100 ns simulation time. The average fluctuation for the APP apo protein, two polyphenols and the control drugs were 1.09 Å, 1.13 Å, 1.09 Å, and 1.30 Å, respectively, for the APP protein, while and at the end, the protein’s amino acid has high points due to presence of terminal carbon groups (Figure 7F). The APP–valiltramiprosate complexes showed a larger peak at VAL214 (6.76 Å), whereas the apigenin and naringenin did not show any significant variation. In the case of CBC, the average fluctuation for the CBS apo, apigenin, naringenin, and control drug benserazide complexes were 1.02 Å, 1.18 Å, 0.98 Å, and 1.33 Å, respectively. The THR 193 (3.12 Å), GLY 246 (2.81 Å), and GLU 297 (2.42 Å), residues from the apigenin complex demonstrated higher peaks, whereas the benserazide complex showed a larger peak at SER63 (12.08 Å), as illustrated in Figure 7G. The CBS apo protein in all of the three compound complexes showed several random peaks from the five to fifty amino acids positions, due to presence of N-terminal at the protein’s beginning site. After that, there were no significant high fluctuations for the rest of the residual positions. In the ETS2 receptors, average RMSF values of 0.75 Å, 0.72 Å, 0.74 Å, and 0.72 Å were observed for the ETS2 apo protein, the two selected polyphenols (apigenin and naringenin), and the TK216 control drug complexes respectively. All the complexes provided multiple rocketed peaks, but the overall fluctuation position below 3.0 Å depicted that the ETS2 protein is also stable when it binds with the apigenin and naringenin lead compounds. High reached peaks at the SER 339, GLY 345, SER 260, ASP387, and LYS 411 residual fluctuations have been generated (Figure 7H). The selected lead complexes from the respective receptors fluctuated in an optimal range with their amino acid positions.

### 2.7. The Radius of Gyration (Rg)

**Figure 8:**
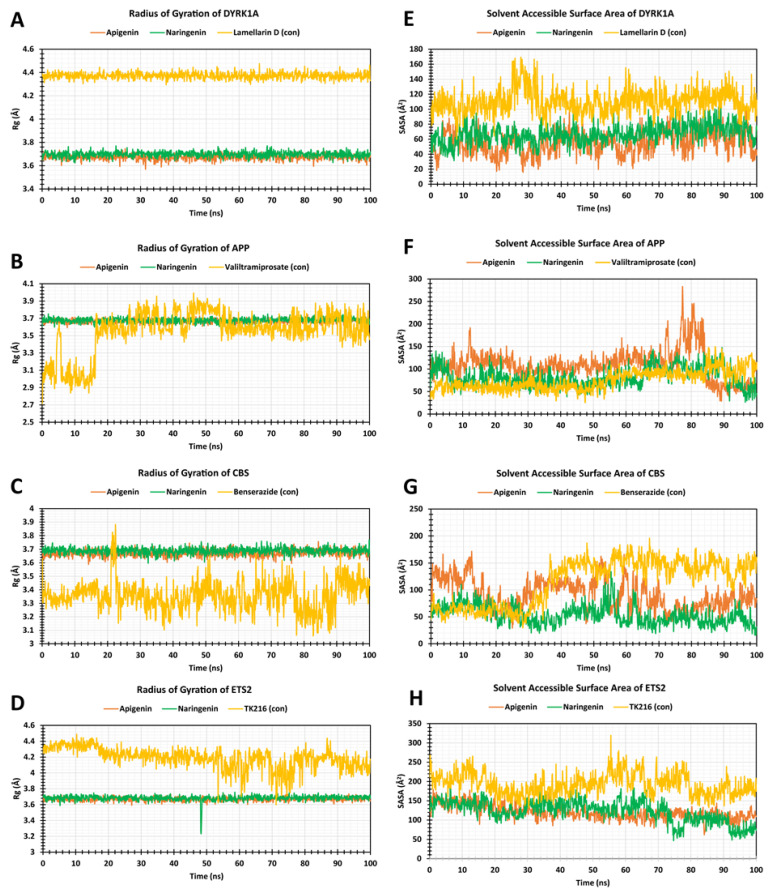
Rg values (Left), and SASA values (Right). Apigenin is shown in orange, naringenin in green and each protein’s respective control drug is shown in yellow. Apigenin and naringenin and the control drug Rg values when complexed with (**A**) DYRK1A, (**B**) APP, (**C**) CBC, and (**D**) ETS2, and SASA values when complexed with (**E**) DYRK1A, (**F**) APP, (**G**) CBC, and (**H**) ETS2.

The two lead compounds, apigenin and naringenin, and the respective protein control drugs’ stability in complex with the DYRK1A, APP, CBC, and ETS2 receptors were calculated based on their Rg values to a 100 ns simulation run time, as outlined in Figure 8A–D. The apigenin in complex with DYRK1A, APP, CBC, and ETS2 receptors showed average Rg values with least and highest Rg fluctuation difference ranges of 3.67 Å (0.177 Å), 3.66 Å (0.151 Å), 3.67 Å (0.169 Å), and 3.66 Å (0.142 Å), while naringenin exhibited 3.69 Å (0.144 Å), 3.67 Å (0.181 Å), 3.68 Å (0.172 Å), and 3.68 Å (0.525 Å) respectively. The average Rg values of DYRK1A, APP, CBs, and ETS2 protein positive control drugs of DYRK1A–lamellarin D, APP–valiltramiprosate, CBS–benserazide, and ETS2-TK216 were 4.37 Å, 3.55 Å, 3.35 Å, and 4.18 Å, respectively, whereas the lowest to highest Rg values were (4.29–4.47) Å, (2.27–3.98) Å, (3.05–3.88) Å, and (3.60–4.48) Å, respectively. In DYRK1A, the two lead compounds, apigenin and naringenin, exhibited lowest Rg values from the 0 ns to 100 ns simulation time. For APP, the apigenin and naringenin showed the very lowest peaks throughout the simulation time, whereas the control drug demonstrated random fluctuation until the 20 ns simulation time. After that, it overlapped with the lead compound complex. In CBS, the control drugs showed lower Rg values than the two lead compounds, but these did not stabilize, due to presenting some major fluctuations from 50 ns to the end of the simulation. Finally, for ETS2, apigenin and naringenin lower Rg values when comparing with the control drugs. The control drugs demonstrated several higher peaks, with greater Rg values. Of all the selected receptors, the apigenin and naringenin demonstrated an acceptable range of Rg fluctuations compared to their respective positive control drugs. The measured Rg value demonstrated better stability in 100 ns simulations, with lower fluctuation, suggesting that apigenin and naringenin with their respective protein complexes significantly improved compactness.

### 2.8. Solvent-Accessible Surface Area

In this study, the apigenin (lead) in complex with DYRK1A, APP, CBC, and ETS2 proteins demonstrated an average fluctuation of 55.31 Å^2^, 109.88 Å^2^, 91.07 Å^2^, and 120.53 Å^2^, respectively. The naringenin showed an average fluctuation of 66.91 Å^2^, 81.88 Å^2^, 53.54 Å^2^, and 122.57 Å^2^, respectively, as shown in in Figure 8E–H. When compared with each protein’s positive control drugs, DYRK1A–lamellarin D, APP–valiltramiprosate, CBS–benserazide, and ETS2–TK216, average SASA values were calculated as 110.42 Å^2^, 74.88 Å^2^, 115.79 Å^2^, and 192.54 Å^2^, respectively. This result indicates that two polyphenols displayed a lower range of surface area exposed to the solvent in complex with DYRK1A, APP, CBC, and ETS2 receptors (Figure 8). In DYRK1A, the positive control drug complex demonstrated a larger peak than the selected ligands from the start to the end of the 100 ns simulation. For APP, the apigenin complex demonstrated several high peaks after the 70 ns simulation, whereas the naringenin showed better stability when compared with the control drugs. For CBS, the selected polyphenols showed stable SASA values, whereas the control drug showed some fluctuations. After that, the SASA values of the apigenin and control drug complexes gradually increased up to 50 ns simulation. Then the lead compounds exhibited stability, but the control drug complex showed larger fluctuations. Finally, for ETS2, the apigenin and naringenin complexes provided better SASA values than those of the of positive control drug from the 100 ns simulation timeframe. This result suggests that there was effective exposure of amino acid residues to the identified compound in the complex systems.

## 3. Discussion

Computer-aided drug design (CADD) has been widely used for more than three decades to screen, develop, and construct molecular candidates with therapeutic significance [37]. These methods are a combination of different approaches including molecular docking, and molecular dynamics (MD) simulation that help to optimize and screen compounds more effectively [38,39]. In current times, molecular docking has emerged as a widely used technique for elucidating the interaction between a target protein and potential small molecule compounds [38]. Initially, the approaches can suggest possible compounds that can be used for drug design [38]. MD simulation can then confirm the stability of these desirable molecules to the target protein. In the past, numerous small molecules have been discovered using CADD methods [40,41]. These molecules have successfully proven effective in lab settings and are now considered innovative therapies for treating Down Syndrome [42,43]. The current study employed network pharmacology to identify the prevalent genes of apigenin and naringenin in DS as prospective targets for therapy. Network pharmacology is a developing field that integrates systems biology and computational methods to understand the interaction networks between medicines and their targets. The binding affinities and interactions of apigenin and naringenin, which are naturally occurring polyphenolic compounds, were examined with important proteins involved in DS, namely DYRK1A, APP, CBS, and ETS2. The findings indicate that these chemicals may have potential therapeutic uses in the management of DS.

DYRK1A, also known as dual-specificity tyrosine-phosphorylation-regulated kinase 1A, plays a critical role in the development of DS because it is excessively expressed in individuals with an extra copy of chromosome 21 [44,45]. The excessive expression of this gene leads to the neurodevelopmental impairments observed in DS [11]. Prior research has shown that suppressing DYRK1A can alleviate cognitive and developmental abnormalities in models of DS [11]. A study emphasized the potential of DYRK1A inhibitors in enhancing cognitive functioning in mouse models with Down Syndrome [25]. The results of our molecular docking analysis are consistent with these findings, indicating that both apigenin and naringenin have high binding affinities to DYRK1A compared with existing positive controls. Naringenin exhibited the most favorable binding score of −9.3 kcal/mol, indicating a strong interaction. These findings indicate that these drugs have the potential to be efficient inhibitors of DYRK1A, which could potentially improve certain neurological symptoms associated with DS. The APP gene, situated on chromosome 21, is excessively expressed in individuals with DS, resulting in heightened synthesis of amyloid-beta and the premature start of AD in DS patients [46,47]. The presence of amyloid-beta plaques is a characteristic feature of AD pathogenesis [48]. Prior studies have demonstrated that polyphenols have the ability to regulate the aggregation of amyloid-beta and diminish its harmful effects on the nervous system [23,49]. Ono et al. (2003) conducted studies that showed polyphenols such as resveratrol have the ability to hinder the aggregation of amyloid-beta and decrease the formation of plaques [50]. Both apigenin and naringenin had significant binding affinities to APP in this study, with apigenin displaying a binding score of −8.8 kcal/mol. This interaction implies that these polyphenols may regulate the activity of APP and the generation of amyloid-beta, thereby decreasing symptoms resembling Alzheimer’s disease in individuals with Down Syndrome [50,51]. This is consistent with previous research on the neuroprotective benefits of polyphenols in models of AD.

CBS is crucial in the function of homocysteine metabolism [52]. CBS dysfunction results in increased homocysteine levels, which are linked to cardiovascular illnesses and cognitive deficits [53,54]. Research has indicated that polyphenols have the ability to impact the process of homocysteine metabolism and enhance cardiovascular well-being [55]. Research reports have emphasized the impact of polyphenols in regulating homocysteine levels and safeguarding against cardiovascular illnesses [55,56]. The results of our study showed that naringenin exhibited a high affinity for CBS, as indicated by a docking score of −8.0 kcal/mol. This indicates that naringenin may assist in regulating homocysteine levels in individuals with DS, hence reducing some of the cardiovascular and cognitive consequences linked to high homocysteine levels. E26 transformation-specific transcription factor 2 (ETS2) plays a role in controlling cell growth and programmed cell death [57,58]. The excessive expression of ETS2 in DS results in aberrant cell growth and heightened programmed cell death, which contributes to the development of several recognized disorders in DS [58]. A research study has demonstrated that manipulating the expression of ETS2 can impact the growth and death of cells in DS models [14]. Our investigation demonstrates that apigenin and naringenin have the potential to modulate the pathways involving ETS2. Both compounds had notable binding affinities, with apigenin displaying a docking score of −7.3 kcal/mol. These interactions have the potential to assist in the management of cellular abnormalities in DS by decreasing aberrant cell growth and death. The molecular docking research yielded comprehensive information regarding the binding affinities and interactions of apigenin and naringenin with the four target proteins. The binding values demonstrated robust affinities, specifically towards DYRK1A and CBS. The investigation of the interaction identified several non-covalent interactions, such as hydrogen bonds, that play a vital role in maintaining the stability and specificity of the protein–ligand complexes. As an illustration, naringenin demonstrated the strongest attraction to DYRK1A, with a value of −9.3 kcal/mol, suggesting a durable and powerful interaction. Subsequently, there was a relationship between it and CBS, which exhibited a notable propensity for binding. Apigenin, however, demonstrated the highest binding score with APP, indicating its potential ability to regulate amyloid-beta levels in DS.

The stability of these interactions over time was confirmed by MD simulations. The RMSD and RMSF indicated that the protein–ligand complexes maintained stability during the whole simulation time [59]. The analysis of the Rg and SASA offered more understanding of the density and external exposure of the complexes, respectively [60]. The RMSD measurements suggest that the complexes reached equilibrium early in the simulation and exhibited little variation afterwards. As an example, the DYRK1A–naringenin complex exhibited an average RMSD of 1.67 Å, indicating consistent and strong binding during the 100-nanosecond simulation. Similarly, the complex formed by the APP protein and apigenin showed an average RMSD of 2.57 Å, suggesting that the interactions between them are stable. These findings indicate that the interaction between apigenin and naringenin with the target proteins is durable, which is essential for their potential therapeutic benefits. The RMSF values identified the regions of flexibility within the proteins, which are crucial for understanding the dynamic behavior of the complexes. The DYRK1A–naringenin complex exhibited an average RMSF value of approximately 1.00 Å. Notably, certain residues such as GLN 320 and LYS 412 displayed greater fluctuations. These variations offer valuable information about the protein’s adaptability and possible structural alterations when a ligand binds to it. The Rg values provide valuable insights into the degree of compaction shown by the protein–ligand complexes. The DYRK1A–naringenin complex displayed a consistent and condensed shape during the simulation, as evidenced by the average Rg value of 3.69 Å. Similarly, the complex formed by the interaction of APP with apigenin displayed an average Rg value of 3.66 Å, indicating that these complexes remain structurally intact over a period of time.

The SASA values quantified the degree to which the protein–ligand complexes were exposed to the solvent on their surface. The DYRK1A–naringenin complex exhibited an average solvent-accessible surface area (SASA) value of 66.91 Å^2^, suggesting a stable and limited surface exposure. This indicates that the chemicals have a strong ability to attach to target molecules and have the potential to be easily absorbed and utilized by a living organism. The APP–apigenin complex exhibited an average SASA value of 109.88 Å^2^, suggesting a slightly increased surface exposure while remaining within a stable range. The results of this study have important implications for the treatment of DS. Apigenin and naringenin have been identified as possible modulators of crucial proteins implicated in DS, which establishes a foundation for more preclinical and clinical research. These compounds have the potential to be developed as part of a comprehensive therapeutic strategy to effectively manage the intricate pathology of DS.

## 4. Materials and Methods

### 4.1. Protein–Protein Interaction Analysis

In this study, the species was designated as *Homo sapiens* with a confidence score of 0.7 and the isolated networks for the DYRK1A, APP, CBC, and ETS2 were removed. The network was visualized by the STRING database (V9.1, Swiss Institute of Bioinformatics Country, Lausanne, Switzerland).

### 4.2. Protein Preparation

The three-dimensional structures of DYRK1A (PDB ID: 5AIK), APP (PDB ID: 4JPE), CBC (PDB ID: 1JBQ), and ETS2 (PDB ID: 4BQA) were obtained from the RCSB Protein Data Bank (PDB). The protein structures were prepared by removing water molecules, metal ions, and co-factors. Nonpolar hydrogen atoms were merged and polar hydrogen atoms were added, and charges were calculated using AutoDock Tools.

### 4.3. Ligand Retrieval and Preparation

Polyphenol compounds, specifically apigenin and naringenin, were identified through an extensive literature review as potential inhibitors of Down Syndrome. The positive control drugs lamellarin D, valiltramiprosate, benserazide, and TK216, respectively, were selected for DYRK1A, APP, CBC, and ETS2. These polyphenols and positive control compounds were retrieved from the PubChem database. The ligands were prepared by detecting aromatic carbons, setting the torsion tree, assigning correct AutoDock 4 atom types, and merging nonpolar hydrogens.

### 4.4. Active Site Identification and Receptor Grid Generation

The active site of each protein, defined as the region that binds with specific molecular substrates to catalyze chemical reactions, was determined. The binding sites identified were used to generate receptor grids for molecular docking using the PyRx virtual screening tool AutoDock Vina.

### 4.5. Molecular Docking

Molecular docking studies were performed using the PyRx AutoDock Vina wizard to predict the best binding modes between the small molecules (e.g., drugs) and the target macromolecules (e.g., proteins). Default configuration parameters were used, and the binding energy (kcal/mol) was calculated. Complexes with the highest binding energy were selected for further evaluation. The binding interactions of the protein–ligand complexes were analyzed using Maestro Schrodinger.

### 4.6. Molecular Dynamics Simulation

A 100 ns molecular dynamics (MD) simulation was performed using Desmond v6.3 in Schrödinger 2020-3 on a Linux environment to evaluate the stability of the protein–ligand interactions. The simulation specifically examined the complexes generated by four target proteins and two polyphenols, apigenin and naringenin. The TIP3P water model was used, using an orthorhombic box shape to preserve a distance of 10 Å from the center. Sodium (Na+) and chloride (Cl^−^) ions were introduced to the solution in order to achieve neutralization, with a salt concentration of 0.15 M. The OPLS3e force field was used. The system was reduced in size while maintaining a constant pressure (NPT) ensemble at 101,325 Pascals and a temperature of 300 K. The stability and dynamic properties of the complexes were assessed by measuring the RMSD, RMSF, Rg, and SASA values.

## 5. Conclusions

This study emphasizes the possible application of apigenin and naringenin as therapeutic medicines for DS by specifically targeting crucial proteins implicated in the disease pathogenesis. The results of molecular docking and MD modeling establish an excellent foundation for further exploration of their therapeutic capabilities. The results align with previous research on the neuroprotective and therapeutic benefits of polyphenols, endorsing their utilization as a component of a multi-targeted strategy for controlling DS. Moreover, the persistence of these molecules in attaching to important proteins like DYRK1A and APP emphasizes their possible efficiency in altering processes important for DS pathophysiology. In vivo studies and clinical trials should be the main emphasis to confirm these conclusions and evaluate the safety and effectiveness of apigenin and naringenin in DS patients. Including these polyphenols into therapeutic strategies could provide a fresh way to control the complicated DS symptoms and lead to better cognitive and neurological results for affected people.

## Figures and Tables

**Table 1:** Molecular docking scores of the selected two polyphenols (apigenin and naringenin) and positive control drugs with four proteins.

S. No.	Receptor Name	Docking Score (kcal/mol)					
		Apigenin	Naringenin	Lamellarin D	Valiltramiprosate	Benserazide	TK216
1	DYRK1A	−9.2	−9.3	−8.9	-	-	-
2	APP	−8.8	−8.6	-	−5.5	-	-
4	CBS	−7.8	−8.0	-	-	−6.4	-
5	ETS2	−7.3	−7.2	-	-	-	−7.1

**Table 2:** Molecular interaction of apigenin and naringenin with four targeted receptors.

Receptor	Compound Name	Interacting Bonds
DYRK1A (Dual-Specificity Tyrosine-Phosphorylation-Regulated Kinase 1A)	Apigenin	LEU164, ILE165, GLY166, LYS167, PHE170, VAL173, LYS175, VAL185, ALA186, ILE187, LYS188, GLU203, LEU207, VAL222, LEU236, PHE238, GLU239, MET240, LEU241, SER242, TYR243, ASN244, ASP247, HIS285, LEU294, LEU295, CYS296, ILE305, VAL306, ASP307, PHE308, GLY309, SER310
	Naringenin	ILE165, GLY166, LYS167, PHE170, VAL173, VAL174, LYS175, VAL185, ALA186, ILE187, LYS188, GLU203, LEU207, VAL222, LEU224, LEU236, PHE238, GLU239, MET240, LEU241, SER242, TYR243, ASN244, ASP247, LEU294, LEU295, CYS296, ILE305, VAL306, ASP307, PHE308, GLY309
	Lamellarin D	ILE165, GLY166, LYS167, GLY168, PHE170, VAL173, ALA186, LYS188, GLU203, LEU207, VAL222, LEU236, PHE238, GLU239, MET240, LEU241, SER242, TYR243, ASN244, ASP247, GLU291, ASN292, LEU294, VAL306, ASP307, PHE308, GLY 309
APP (Amyloid Precursor Protein)	Apigenin	LEU78, ASP80, GLY82, SER83, SER84, ASN85, PHE86, ALA87, VAL88, VAL117, PRO118, TYR119, THR120, GLN121, GLY122, LYS123, TRP124, ALA148, ILE150, ASP154, LYS155, PHE156, PHE157, ILE158, TRP163, GLY165, ILE166, LEU167, GLY168, ILE174, ALA175, ARG176, GLY278.
	Naringenin	LEU78, ASP80, GLY82, SER83, SER84, ASN85, PHE86, ALA87, VAL88, VAL117, PRO118, TYR119, THR120, GLN121, GLY122, LYS123, TRP124, ALA148, ILE150, ASP154, LYS155, PHE156, PHE157, ILE158, TRP163, GLY165, ILE166, LEU167, GLY168, ILE174, ALA175, ARG176, GLY278
	Valiltramiprosate	LEU78, ASP80, GLY82, SER83, SER84, ASN85, PHE86, ALA87, VAL117, PRO118, TYR119, THR120, GLN121, GLY122, TRP124, ILE150, PHE156, ILE166, LEU167, GLY168, ILE174, ALA175, ARG176, ASP276, GLY278
CBS (Cystathionine Beta-Synthase)	Apigenin	THR68, ALA69, PRO70, ALA71, LYS72, SER73, PRO74, LEU77, PRO78, LEU81, LYS82, LYS83, ILE84, GLY85, ASP86, ASN113, VAL118, LYS119, ASP120, ARG121, ILE122, SER123, LEU124, ARG125, MET126, GLU128, ARG161, SER227, ASN228, LEU230, ALA231, HIS232, ASP234, THR235, THR236, GLY239
	Naringenin	THR68, ALA69, PRO70, ALA71, LYS72, SER73, PRO74, LEU77, PRO78, LEU81, LYS82, LYS83, ILE84, GLY85, ASP86, THR87, LYS108, GLU110, ASN113, VAL118, ASP120, ARG121, ILE122, SER123, LEU124, ARG125, MET126, ILE127, GLU128, ASP129, ARG161, SER227, ALA231, HIS232, ASP234, THR235, THR236, GLU239
	Benserazide	THR68, PRO70, ALA71, LYS72, SER73, PRO74, LEU81, LYS82, LYS83, ILE84, GLY85, ASP86, THR87, ASN113, ASP120, ARG121, ILE122, LEU124, ARG125, GLU128, SER227, LEU230, ALA231, HIS232, THR235, THR236, GLU239
ETS2 (E26 Transformation-Specific Transcription Factor 2)	Apigenin	LEU369, LEU370, GLU371, LEU372, LEU373, HER374, ASP375, LYS376, SER377, CYS378, GLN379, SER380, PHE381, ILE382, SER383, TRP384, THR385, GLY386, ASP387, GLY388, GLU390, LYS392, PRO454, GLU455, LEU457, HIS458, ALA459, ILE460
	Naringenin	LEU369, LEU370, GLU371, LEU372, LEU373, HER374, ASP375, LYS376, SER377, CYS378, GLN379, SER380, PHE381, ILE382, SER383, TRP384, THR385, GLY386, ASP387, GLY388, GLU390, LYS392, PRO454, GLU455, LEU457, HIS458, ALA459, ILE460
	TK216	ALA355, THR358, GLY359, SER360, GLY361, PRO362, ILE363, GLA364, LEU365, TRP366, GLN367, PHE368, LEU369, LYS392, ALA400, TRP403, ARG406, LYS407, ASN408, LYS409, PRO410, LYS411, MET412, ASN413, TYR414, GLU415, LYS416, LEU417, SER418, ARG419, GLY420, LEU421, TYR423, TYR424, TYR425, ASP426, LYS427, ILE429, CYS444

## Data Availability

The data generated from the study has been clearly presented and discussed in the manuscript.

## Supplementary Materials

The following supporting information can be downloaded at: https://www.mdpi.com/article/10.3390/ph17081090/s1, Figure S1: A merged image of apigenin and naringenin with their respective target proteins to facilitate a direct comparison of their binding modes. This visual representation should provide (A) DYRK1A, (B) APP, (C) CBS, and (D) ETS2 protein’s active site. Here, apigenin showed red color and naringenin showed green color, respectively.

## Abbreviations

DS (Down Syndrome), AD (Alzheimer’s disease), APP (amyloid precursor protein), CBS (cystathionine beta-synthase), ETS2 (E26 transformation-specific transcription factor 2), RMSD (root mean square deviation, RMSF (root mean square fluctuation), Rg (radius of gyration), SASA (solvent-accessible surface area).
