# Supplementary material for: Network Pharmacology Identifies Intersection Genes of Apigenin and Naringenin in Down Syndrome as Potential Therapeutic Targets
Source: Pharmaceuticals (Basel). 2024 Aug 20;17(8):1090. doi: 10.3390/ph17081090 (PMC11359399; doi:10.3390/ph17081090)
Supplement: Supplementary file 1 [file pharmaceuticals-17-01090-s001.zip › pharmaceuticals-3126483-supplementary.pdf]

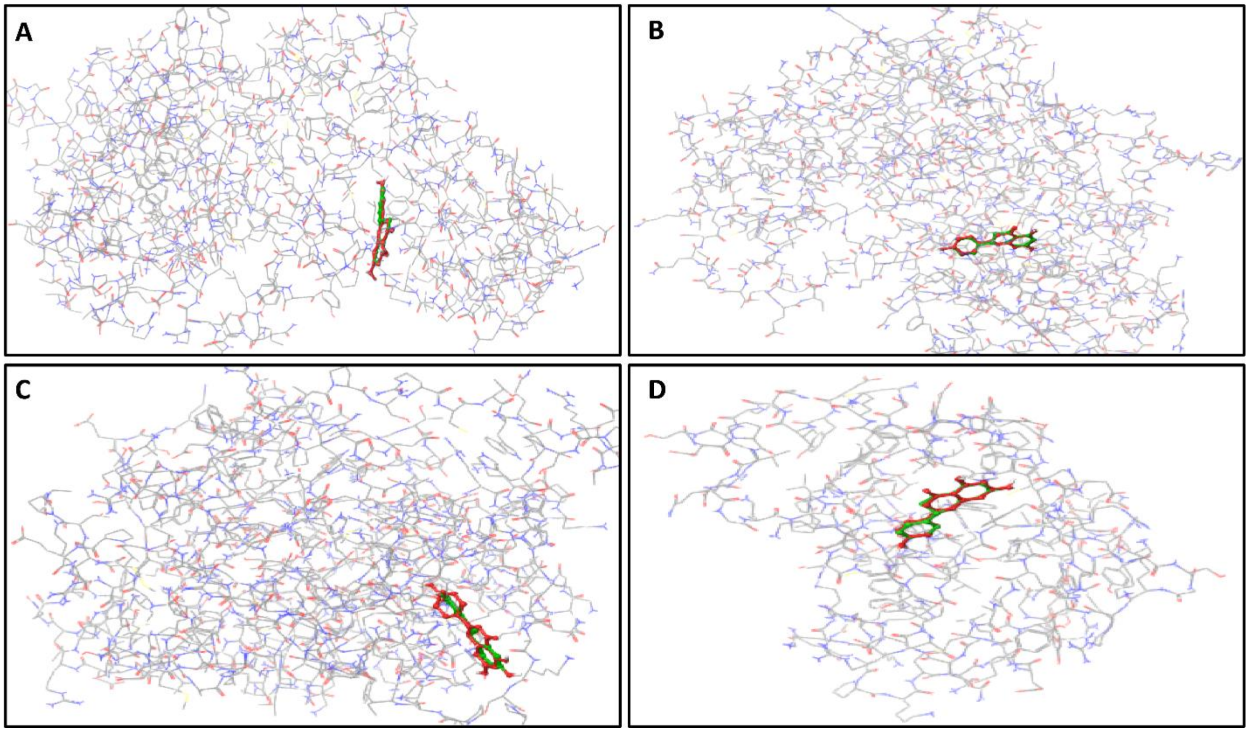

Supplementary Figure S1: A merged images of apigenin and naringenin with their respective target proteins to facilitate a direct comparison of their binding modes. This visual representation should provide (A) DYRK1A, (B) APP, (C) CBS, and (D) ETS2 protein's active site. Here, apigenin showed red color and naringenin showed green color, respectively.
